# Striatal dual cholinergic /GABAergic transmission in Parkinson disease: friends or foes?

**DOI:** 10.15698/cst2018.06.142

**Published:** 2018-05-27

**Authors:** Natalia Lozovaya, Yehezkel Ben-Ari, Constance Hammond

**Affiliations:** 1 Neurochlore and B&A Therapeutics, Ben-Ari Institute of Neuroarcheology, Batiment Beret-Delaage, zone Luminy entreprises, 13288 Marseille, Cedex 09, France.

**Keywords:** ACh/GABA cotransmission, striatum, GABA polarity, Parkinson disease

## Abstract

The rule of one terminal and one transmitter acting on one synapse clearly fails to cover the complexity of chemical synapse operation in the brain. Compelling evidence now indicates that two transmitters can be released from the same terminal, acting in a complementary manner to generate complex electrical activity in the targets. Our laboratory now showed that a subpopulation striatal cholinergic neurons also release the classical inhibitory transmitter GABA with a balance between excitation and inhibition being provided by acetylcholine and GABA, respectively. An illustration of the importance of this dual release comes from the fact that when dopamine signals are absent such as in Parkinson disease (PD) the GABAergic inhibition in these dual cholinergic/GABAergic cells fails because of high intracellular chloride ((Cl^-^)_I_) levels rendering the cholinergic excitatory component unmet by a parallel inhibitory drive. Restoring low (Cl^-^)_I_ with the NKCC1 chloride importer antagonist bumetanide attenuates the electrical and motor disturbance. In addition to illustrating the complex interactions between two transmitters acting at the same synapse, this study paves the way to novel conceptual treatment of PD based on restoration of GABAergic inhibition in keeping with our pilot clinical trial showing indeed that bumetanide together with levodopa attenuates axial motor disturbance. It is also in keeping with extensive investigations showing increased (Cl^-^)_I_ levels and weakened inhibition in a wide range of pathological insults and their restoration by bumetanide. It raises fundamental issues related to the operation of the striatum and basal ganglia in health and disease.

Although constituting less than 1% of neuronal population of the striatum, cholinergic interneurons play a major role in the control of sensory integration in preparation for movements. These giant neurons innervate a wide range of targets and are in a capacity to modulate the output of the striatum and the operation of the basal ganglia. In our study, we noted that half of these cholinergic interneurons are also positively labeled by Lhx6 a transcription factor that labels some GABAergic neurons; hence, our quest was to test the possibility that these cholinergic neurons are also GABAergic. To fully demonstrate that, we relied on immune-histochemical tests and intracellular RT-qPCR techniques showing mRNAs coding for cholinergic and GABAergic markers but also Lhx7 and GAD, thus confirming their dual features. Optogenetic and/or direct stimulation in paired recordings of these mixed Cholinergic GABAergic interneurons (CGINs) generate mixed GABAergic/ cholinergic PSCs in other CGINs. The classical pause response evoked by cortical stimulation is highly dependent on (Cl^-^)_I_ levels, being abolished when these are elevated stressing the importance of GABA_A_-mediated inhibition (**Figure 1**, top). Using the iDISCO clarification technique, we found that up to 50% of cholinergic interneurons in the dorsal striatum are dual CGINs stressing their numerical importance.

**Figure 1:**
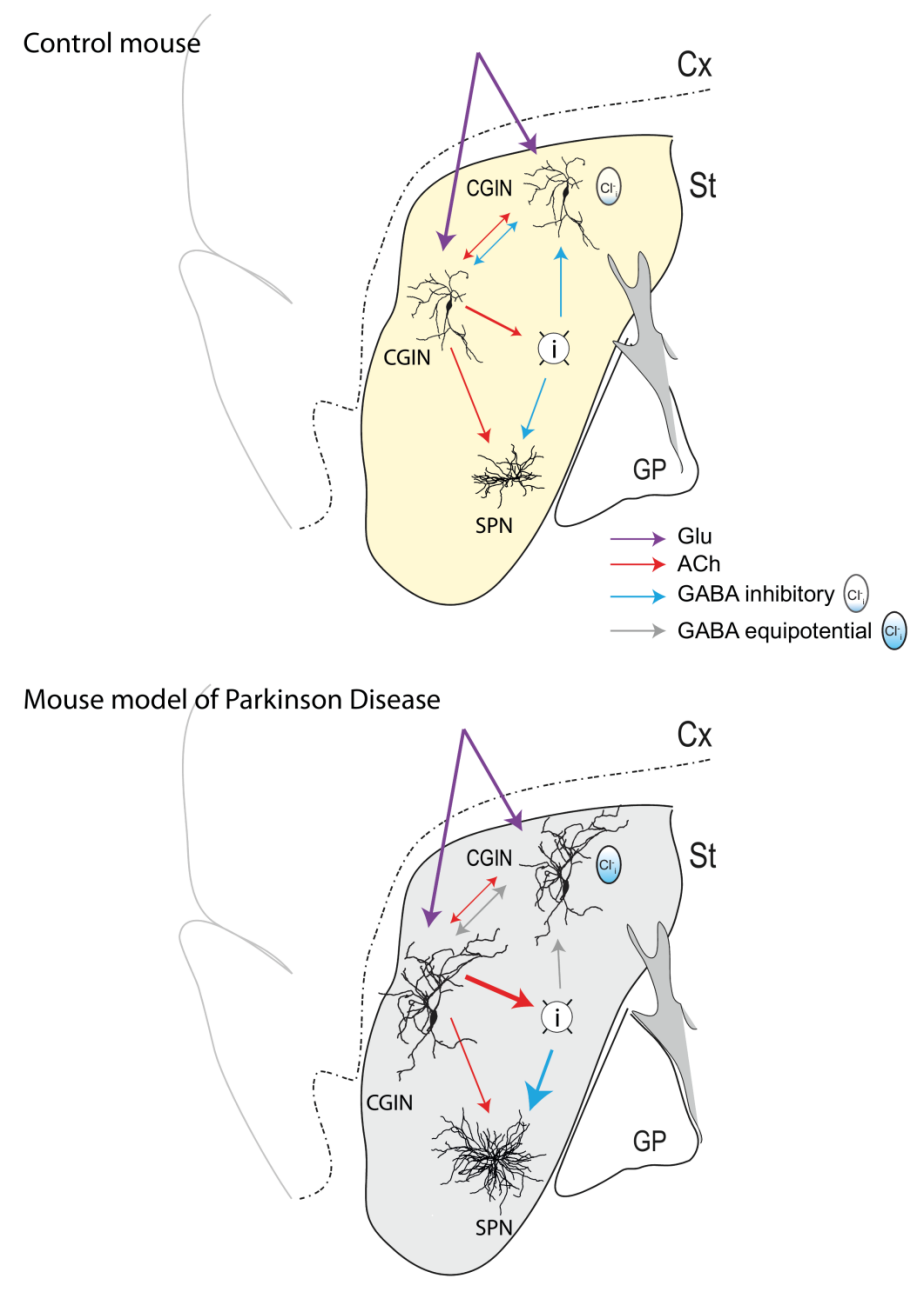
FIGURE 1: Schematic drawing of a mouse brain sagittal section showing sensory motor cortex (Cx), dorsal striatum (St), and Globus Pallidus (GP). Yellow indicates control dopaminergic innervation (top) whereas gray indicates depletion of dopaminergic terminals (bottom). Connections between CGINs, GABAergic Interneurons (I) and Spiny Projections Neurons (SPN) with different colors as indicated. Polarity of GABA actions depend on the intracellular chloride levels as illustrated (icon).

Perhaps the crux and major novelty of this study is the demonstration that this dual response is profoundly altered in a rodent model Parkinson Disease (PD). Indeed, dopamine deprivation abolishes the GABAergic inhibitory component of this dual PSC leaving the excitatory cholinergic drive unmet by an inhibitory control (**Figure 1**, bottom). This abolition is due to high (Cl^-^)_I_ levels as restoring low (Cl^-^)_I_ levels either by whole-cell patch recording or by ad-ministration of the NKCC1 chloride importer antagonist bumetanide -a classical reducer of (Cl^-^)_I_ levels - also restores GABAergic inhibition and the pause response previously abolished by dopamine deprivation. Finally, bumetanide also attenuates the motor disturbances produced by dopamine deprivation. Collectively, these observations suggest that the equilibrium between excitation and inhibition in target neurons of CGINs is instrumental in the integration of information by the striatum and is highly dependent on dopamine signals by mechanisms that await determination.

In a more general perspective, this study raises important questions in a fundamental and therapeutic perspective. Dual Ach/GABA transmission has now been shown to operate in many structures including the globus pallidus, cortex, hippocampus, olfactory system etc. This will most likely be shown to be a widespread feature that has been preserved throughout evolution serving important tasks that are not met by a single transmitter. Indeed, the release of two transmitters implies the concomitant adjustment of plethora of synaptic mechanisms including release mechanisms, pre and postsynaptic receptor channels, transport and other devices to control the duration of the synaptic currents etc. Yet, this complexity apparently offers an advantage in terms of flexibility, widening the range of integrative mechanisms. An important issue to investigate is the developmental origin of these dual release devices. Indeed, GABA has been shown to antecede other transmitters - GABA before glycine in the chord or before glutamate in the hippocampus - raising the possibility of an ancillary transmitter that in some synapses comes first and is replaced later in development whereas in other synapses this duality remains for reasons that remains to be understood.

In a therapeutic perspective, it is important to stress that depolarizing/excitatory GABA has been shown to operate in a wide range of disorders extending from epilepsies, to autism, Rett syndrome, spinal cord lesion and brain trauma, suggesting that the failure of (Cl^-^)_I_ levels regulation and its accumulation are a universal reaction to insults. In addition, bumetanide in many of these situations restores or at least attenuates the severity of the syndrome, paving the way to novel therapeutic strategies relying on the regulation of (Cl^-^)_I_ levels. In keeping with this, we found in a pilot trial on four patients that bumetanide treatment attenuates some motor symptoms of PD. Yet, perhaps the most complex missing element of the puzzle will be to understand where and how dopamine regulates (Cl^-^)_I_ levels. This will require determination of the interactions between (Cl^-^)_I_ levels regulation and dopamine signals and how the former is hampered in PD. There is little doubt that the demonstration that a pathological insult affecting one component of a dual response will impact our understanding of the roles of dual transmitters and of the interactions between the two players in health and disease.

These observations raise many questions that might alter our understanding of how basal ganglia operate. The extensive morphological rearrangements also observed earlier in the spiny projecting neurons that constitute the majority of striatal neurons suggests that dopamine deprivation produces a general rearrangement of intrinsic and possibly extrinsic connections, a process that might have to be incorporated in our understanding of the pathogenesis of PD. Likewise, to reconstruct the puzzle, we need to understand whether all synaptic connections -in particular in GABAergic interneurons known to control the activity of SPN - have also altered (Cl^-^)_I_ levels. Where and how does dopamine regulate (Cl^-^)_I_ levels and the efficacy of the GABAergic component in interneurons and notably of the dual PSCs. This will require determination of the interactions between (Cl^-^)_I_ levels regulation and dopamine signals and how the former is hampered in PD. There is little doubt that the demonstration that a pathological insult affects one component of a dual response will impact our understanding of the interactions between the two players in health and disease.

In summary, we found that the GABAergic component of the dual excitatory Ach/ inhibitory GABA transmission of striatal cholinergic neurons is altered in PD because of elevated (Cl^-^)_I_ levels and attenuated inhibition. Restoration of low (Cl^-^)_I_ levels and an efficient GABAergic inhibition attenuates electrical and behavioral sequels of dopamine signals deprivation. Results call for incorporating the alterations of inhibition in a dual transmitter system in PD and other degenerative disorders.

